# Metal 3D-Printed Bioinspired Lattice Elevator Braking Pads for Enhanced Dynamic Friction Performance

**DOI:** 10.3390/ma17112765

**Published:** 2024-06-05

**Authors:** Nikolaos Kladovasilakis, Eleftheria Maria Pechlivani, Ioanna K. Sfampa, Konstantinos Tsongas, Apostolos Korlos, Constantine David, Dimitrios Tzovaras

**Affiliations:** 1 Centre for Research and Technology Hellas, Information Technologies Institute (CERTH/ITI), 57001 Thessaloniki, Greece; riapechl@iti.gr (E.M.P.); dimitrios.tzovaras@iti.gr (D.T.); 2 R&D Department, KLEEMANN Group, 61100 Kilkis, Greece; i.sfampa@kleemannlifts.com; 3 Advanced Materials and Manufacturing Technologies Laboratory, Department Industrial Engineering and Management, School of Engineering, International Hellenic University, 57001 Thessaloniki, Greece; ktsongas@ihu.gr (K.T.); apkorlos@ihu.gr (A.K.); 4 Manufacturing Technology and Production Systems Laboratory, Department of Mechanical Engineering, International Hellenic University, 62124 Serres, Greece; david@ihu.gr

**Keywords:** bioinspired surface lattices, additive manufacturing, selective laser melting, dynamic friction, elevator’s braking pads, tribological analysis, finite element models

## Abstract

The elevator industry is constantly expanding creating an increased demand for the integration of high technological tools to increase elevator efficiency and safety. Towards this direction, Additive Manufacturing (AM), and especially metal AM, is one of the technologies that could offer numerous competitive advantages in the production of industrial parts, such as integration of complex geometry, high manufacturability of high-strength metal alloys, etc. In this context, the present study has 3D designed, 3D printing manufactured, and evaluated novel bioinspired structures for elevator safety gear friction pads with the aim of enhancing their dynamic friction performance and eliminating the undesired behavior properties observed in conventional pads. Four different friction pads with embedded bioinspired surface lattice structures were formed on the template of the friction surface of the conventional pads and 3D printed by the Selective Laser Melting (SLM) process utilizing tool steel H13 powder as feedstock material. Each safety gear friction pad underwent tribological tests to evaluate its dynamic coefficient of friction (CoF). The results indicated that pads with a high contact surface area, such as those with car-tire-like and extended honeycomb structures, exhibit high CoF of 0.549 and 0.459, respectively. Based on the acquired CoFs, Finite Element Models (FEM) were developed to access the performance of braking pads under realistic operation conditions, highlighting the lower stress concentration for the aforementioned designs. The 3D-printed safety gear friction pads were assembled in an existing emergency progressive safety gear system of KLEEMANN Group, providing sufficient functionality.

## 1. Introduction

The elevator industry is constantly expanding, driven by the development of cities and the increasing demand for high-rise buildings [1,2]. Concurrently, the imperative for safer and more efficient vertical transportation in buildings of all types persists. Technological advancements in this sector enable the development of elevators with superior performance and safety standards. Adherence to safety regulations is paramount in both the construction and operation phases of elevators. Mechanical components, such as emergency braking systems and safety gears, play a critical role in elevator safety and are required by national and European regulations [3,4].

**Figure 1:**
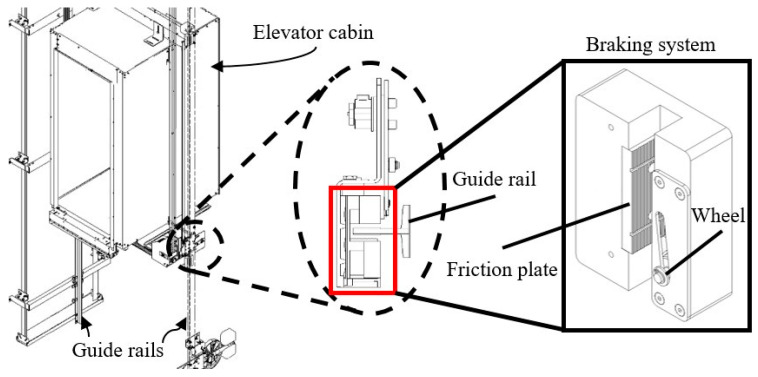
Image of an indicative existing progressive emergency braking system along with its position on the elevator’s system.

Progressive safety gear is a mechanical device used to deal with situations of potential danger, such as exceeding the descent speed or breaking the means of suspension, for example, wire ropes [5,6]. In these cases, an innovative grab is activated automatically, ensuring the immediate stop of the elevator cabin movement and protecting the occupants from possible accidents. In detail, this progressive safety gear can be placed both under the cabin, to avoid sudden downward and/or upward movement, and under the counterweight, in the event that the lower space in the shaft, and in particular under the travel of the counterweight, is accessible. The basic elements of the progressive safety gear include the friction plate (pad), the housing, disc springs, and the gripper wheel. In order to avoid over-acceleration of the elevator, especially for elevators with a nominal speed of more than 1 m/s, the progressive safety gear is used according to the state of the art. Figure 1 shows an indicative image of an existing progressive safety gear system along with its position on the elevator’s system. Specifically, the following phases can be distinguished in the operation of the progressive safety gear:

- The resting phase, where the wheel is at a safe distance (a few millimeters) from the guide rails, while the friction plate is at a short distance of 1–3 mm from the guide rails.
- Activation phase, where the wheel has just touched the guide rails
- The wedging phase is where the wheel moves and comes into contact with the guide rails until the gap between the guide rails and the back is zero, at which point the safety gear begins to brake.
- After the guide rails are in contact with the friction plate, the springs are compressed and thus the friction forces develop gradually, resulting in progressive braking.

The main disadvantages of existing commercial safety gears in the elevator progressive emergency braking system are the damages caused both on the safety gear itself and the guide rails during activation. More specifically, when the safety gear of an elevator is activated, the friction plates strongly deform the guide rails’ surfaces leading to complete failure after one or at most two times of emergency braking, forcing their replacement. These incidents increase the cost of maintenance and the operational costs of the elevator due to the downtime period, which in many cases is highly undesirable, especially in buildings where the elevator system is a vital infrastructure, such as in hospitals. In addition, with the existing morphology of the commercial friction plates, it becomes difficult to predict the behavior of the material, since instead of achieving immobilization of the cabin (or counterweight) through the frictional forces developed; the results often are the plastic deformation of the guide rails due to partial penetration of the friction plate into guide rails body. This behavior also leads to an undesirable experience for the passengers of the elevator, increasing the possibility of their injury. In order to address the aforementioned issues, in recent decades, both the research community and the elevator industry examined and developed novel solutions optimizing the performance and reliability of the progressive safety gear. There are numerous submitted patents in this direction. However, there is a lack of published literature about the examination of innovative patterns in the contact area of a friction plate to increase its dynamic frictional performance.

**Figure 2:**
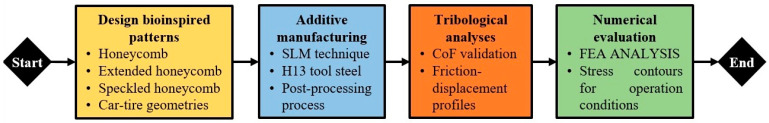
Flowchart of the current study.

In this context, the current study proposes the technical solution of integrating hierarchical biomimetic patterns on the surface of a friction pad in order to develop and manufacture pioneering friction pads, with optimal production techniques and advanced materials. The employment of this novel approach is expected to provide a smoother immobilization of the elevator, reducing the possibility of injury of the passengers, and avoiding the unpleasant experience of a sudden and emergency elevator stop. In addition, it is anticipated to increase the lifetime of the specific components, due to the existence of large diffusion canals, reducing the maintenance intervals and costs of an elevator. More specifically, in this paper, the development of four innovative friction plates for the safety gear of the elevator emergency braking system were developed. These advanced morphologies were based on the application of bio-inspired hierarchical patterns, i.e., specific repeated geometric shapes, which can significantly improve the tribological behavior of a surface. This innovation of hierarchical patterns design can be incorporated into various types of elevator safety gear offering comparative advantages. In detail, the superiority of these new morphologies of the present study is in the application of a biomimetic friction surface design process, i.e., in copying the morphology of natural patterns with proven high adhesion, such as the sole of a frog’s rear ends. The developed morphologies are the honeycomb, extended honeycomb, speckled honeycomb, and car-tire geometries with a biomimetic design. The designed friction pads were additively manufactured using the Selective Laser Melting (SLM) technique and a tribological analysis was performed for each one. Then, based on the results of tribological analyses, i.e., coefficient of friction (CoF), the produced braking pads were numerically evaluated via Finite Element Analyses (FEAs) under realistic loads. Figure 2 presents the flowchart of the current study.

## 2. Materials and Methods

### 2.1. Problem Statement

Progressive safety gears are widely utilized in the elevator industry, increasing the safety and reliability of elevator systems. Typically, the progressive safety gear of a commercial elevator consists of two friction pads with a trapezoidal configuration to achieve progressive deceleration. In this way, the immobilization of the elevator occurs smoothly avoiding potential injuries to the passengers. However, in order to achieve the immobilization of an elevator, there is a need to create a friction force (opposite to the movement of the elevator) on the guide rails greater than its overall weight force. According to existing standardization [7], the overall weight force of an elevator is provided by the following equation (Equation (1)). Where *P* is the mass of the empty cabin which includes the masses of doors, walls, floor, roof, and cabin sling. On the other hand, *Q* is the payload of an elevator, which is the maximum weight it can carry for the selected cabin [8]. Furthermore, *g* is the gravitational acceleration (9.81 m/s^2^) and *FOS* is the factor of safety which ensures that deceleration of the elevator occurs. The value of this property is usually around 2.

(1) $$Total\;Braking\;Force=\left(P+Q\right)\times g\times FOS$$

Therefore, the braking force (*BF*) for each safety gear is evaluated by the following equation. This equation indicates the necessary vertically applied force (*VF*) from the friction pads to guide rails in order to achieve the desired friction force (Equation (3)). Where *μ* is the experimental coefficient of friction between the friction pad and the guide rail.

(2) $$BF=\frac{Total\;Braking\;Force}{2}$$

(3) $$VF=\frac{BF}{\mu }$$

From the aforementioned analysis, it is obvious that in order to immobilize an elevator, a sufficient amount of vertical force should be applied on the guide rails, much higher than the weight force, due to the fact that the coefficient of friction ranges between 0 and 1. This amount of vertical force often severely damages the guide rails, increasing dramatically the maintenance cost after an emergency brake activation. Therefore, elevator manufacturers explore novel ways to reduce the amount of vertical force. The most straightforward technique to reduce the vertical force is to increase the coefficient of friction between the friction pads and the guide rails. According to the datasheet of the KLEEMANN Group elevator manufacturer, the existing friction pads exhibit a coefficient of friction of around 0.3. Hence, in the scope of this paper, novel surface architected materials are employed on the surface of the friction pad in order to increase the coefficient of friction and thereby reduce the applied vertical force offering efficient and smooth elevator immobilization without damaging its guiding system.

### 2.2. Design of Bio-Inspired Braking Pads

**Figure 3:**
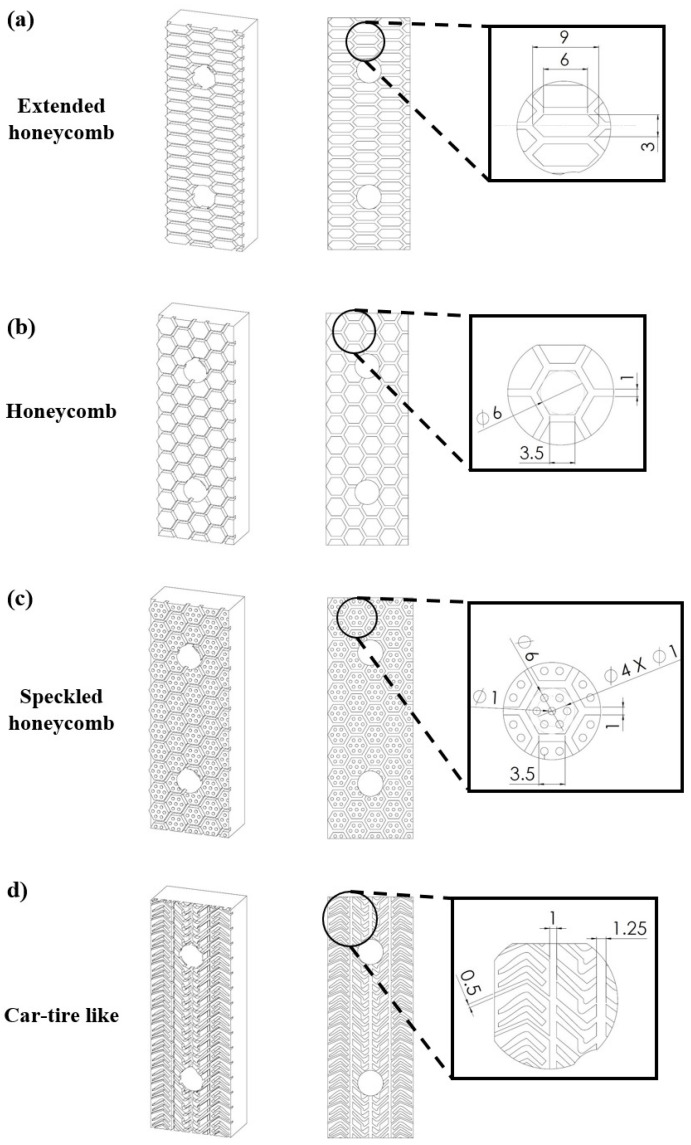
Isometric, front, and detailed view of the designed surface architected materials of (unit: mm): (**a**) Extended honeycomb; (**b**) Honeycomb; (**c**) Speckled honeycomb; (**d**) Car-tire-like configurations.

In the context of this study, four different surface-architected materials were employed with the aim of increasing the surface’s coefficient of friction. These structures were inspired by natural structures with high dynamic friction performance, such as the pads of a frog’s back legs and the pads of cheetah legs for honeycomb structures and for car-tire-like structures, respectively [9,10]. The selected surface-architected materials, i.e., extended honeycombs, honeycombs, speckled honeycombs, and a car-tire-like structure, were integrated into the structure of existing friction pads as it is presented in Figure 3. In general, honeycomb configurations are employed in the automotive industry [11] for braking applications showing enhanced braking performance, therefore, three of the four chosen structures belong to this category. On the other hand, the car-tire structure has been designed to have traction on the road, thus the corresponding structure (car-tire-like) was a suitable candidate for an elevator braking pad. The concept behind the selection of these structures was to create consecutive extended contact surfaces with sufficient channels for debris removal. In this way, the accumulated friction increases the coefficient of friction between the pad and the smooth surface of the guide rail minimizing the damage due to the reduced vertical force and the uniform removal of metal filings during the braking process. The designed dimensions of the employed structures were chosen in order to offer high printability for the developed friction pads and ensure sufficient diffusion channel width. The thickness of each structure’s elements (i.e., for unit cell) was 2 mm in order to provide an extended life cycle (i.e., more than one use) for the braking pad without compromising their mechanical performance. It is worth mentioning that the design process was performed in the SolidWorks™ design platform (Dassault Systèmes SE, Vélizy-Villacoublay, France).

### 2.3. Material Selection and Additive Manufacturing

After the design of friction pads, the next stage is the manufacturing process for these parts. The first step in this process is the material selection. The existing friction pads are fabricated from tool steels in order to withstand the increased braking loads. In this context, for the developed friction pads, the high-strength H13 tool steel was selected as the construction material. The upper section of Table 1 lists the basic properties of the H13 tool steel. These properties were provided by the manufacturer (OC Oerlikon, Freienbach, Switzerland) of the raw material for metal additive manufacturing and from a previously published study on 3D-printed H13 tool steel [12].

Therefore, for the fabrication of the developed braking pads, the SLM additive manufacturing technique was employed utilizing the ORLAS Creator (Coherent Inc., Santa Clara, CA, USA) metal 3D printer. The ORLAS Creator is equipped with a continuous 250 W Yb-fiber laser beam with a wavelength of 1067 nm to sufficiently melt the metal powder. Moreover, its maximum printing accuracy reaches up to 25 μm at the vertical direction/z axis (layer height) and 40 μm in the XY plane (hatching distance) [14]. The selected material was in the form of fine powder and its basic characteristics of particle size distribution (PSD), Hall flow, and chemical composition are listed in Table 1. This combination of parameters ensured the high printability and quality of the 3D-printed friction pads. Furthermore, in order to achieve the optimum quality in terms of mechanical behavior and accuracy, specific process-related parameters were selected based on the results of previous studies (Bottom side of Table 1). It is worth noting that Equation (4) describes the mathematical relation between one of the most important parameters for the fusion of the powder, i.e., the volumetric energy density (*VED*), with the basic process-related parameters, such as layer height (*l*), hatching distance (*h*), laser power (*P*), and printing speed (*V*). Based on the value of VED, we ensured the proper thermal energy exposure for the powder in order to create sufficient and uniform molten pools, which increase the relative density of the part (>99.8%) and eliminate the potential defects (un-melted particles, keyholes, voids, etc.) [15]. Finally, printing orientation was chosen to be ZXY, according to international standards [16], in order for the printed parts to present the maximum strength under operation conditions.

(4) $$VED=\frac{P}{V\cdot h\cdot l}$$

### 2.4. Tribological Analysis

The first step before conducting the tribological analysis was the roughness measurements of the surface of the 3D-printed parts. The surface roughness analysis was performed using the Wyko^®^ NT Series optical profiler (Veeco Instruments Inc., Plainview, NY, USA), NT1100 Optical Profiling System. This system employs white light interferometry for high-resolution 3D surface roughness measurements. Specimens’ profiles were assessed utilizing the optical surface profilometer, specifically focusing on the friction surface of the 3D-printed H13 pads. Discussing surface roughness alongside tribological results is important because it directly influences friction and wear behavior, providing crucial insights into material interactions and performance. It is worth noting that the most crucial roughness parameters for the specimen’s surface and profile were obtained according to international standards [17,18].

In a series of tribological experiments conducted by a Tribolab Universal Material Testing (UMT) apparatus, employing a pin diameter of 6.56 mm, a standardized normal force of 50 N was applied to a distance of 10 mm with a sliding velocity of 1 mm/s. These meticulously controlled parameters served as the foundation for a rigorous examination of frictional dynamics and wear characteristics inherent to diverse material compositions [19,20]. Utilizing the advanced capabilities of the UMT TriboLab from Bruker (Billerica, MA, USA), a pin-on-disc configuration was employed to investigate the tribological behavior of AM H13 steel specimens. These specimens were designed as safety braking pads, featuring various configurations including honeycomb, honeycomb speckled, extended honeycomb, car-tire-like, and pyramid structures. The focus of the investigation centered on determining the CoF, a widely accepted standard parameter of friction. This parameter is calculated from the force sensor signal, considering both the normal and longitudinal forces acquired simultaneously. By analyzing the CoF, insights into the frictional characteristics of each configuration were acquired, facilitating a comprehensive understanding of their tribological performance. This setup facilitated a focused inquiry into the tribological properties of these design patterns examined, aiming to discern their performance under controlled conditions. All tests were performed at least five times at room temperature, on the region between fixation holes.

### 2.5. Finite Element Models

**Figure 4:**
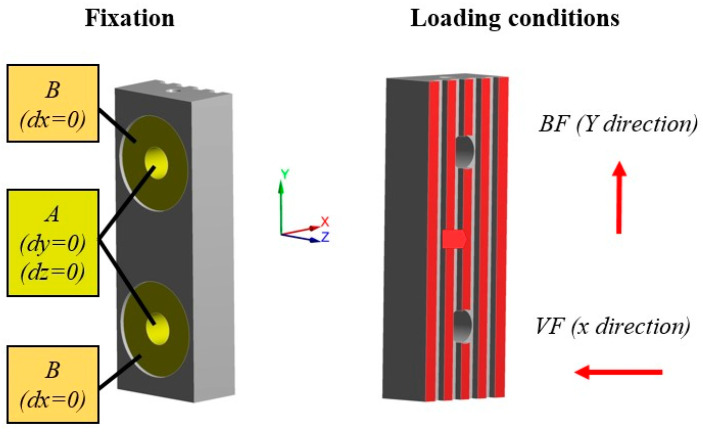
Fixation and loading conditions on the commercial friction pad for the developed FEMs.

In order to evaluate fast, accurately, and reliably the performance of the developed friction pads, finite element models (FEMs) were developed based on the results of tribological analysis. For this purpose, the ANSYS Workbench (ANSYS, Inc., Canonsburg, PA, USA) simulation platform was utilized and, more specifically, its static module. The employed material model was a bilinear isotropic hardening in which the elastic section emerged from the aforementioned material properties and the plastic section occurred after the yield stress point with a tangent modulus of 10,000 MPa based on the experimental results of a previous study [12]. The next step was the creation of the computational mesh, which was defined after the conduction of mesh sensitivity analyses regarding the maximum von Mises stress in order to extract mesh-independent results. This analysis led to mesh with tetrahedral elements and a minimum size of 0.4 mm with total mesh elements ranging from around 100,000 to 125,000 elements depending on the applied geometry of the structure. Regarding the fixation condition of the analyses, each friction pad was set to have zero displacements on the plane of each support hole and zero displacements on the back of each pad where the spring set was applied. On the other hand, for the loading condition, the worst-case scenario of a commercial passenger elevator was chosen with P + Q at 2600 kg and two progressive safety gears. Moreover, the overall force applied to the friction surface of each pad was analyzed in two different components, namely the vertical force and braking/friction force. The braking/friction force has a direction opposite to the movement of the elevator and it is constant due to the fact that the same value for this force results in the immobilization of the elevator regardless of the applied surface pattern of each pad. In contrast, the necessary vertical force in order to create the friction force is dependent on the coefficient of friction of each developed friction pad. Therefore, the evaluation of this force component for each case was performed utilizing the results of the tribological analysis. It is worth mentioning that in the context of this study, a commercial friction pad was also tested in order to provide a comprehensive benchmark for the designed braking pads. Figure 4 presents an indicative example of the commercial friction pad with the fixation and loading conditions that have been considered in the developed FEM.

## 3. Results

### 3.1. 3D-Printed Friction Pads

**Figure 5:**
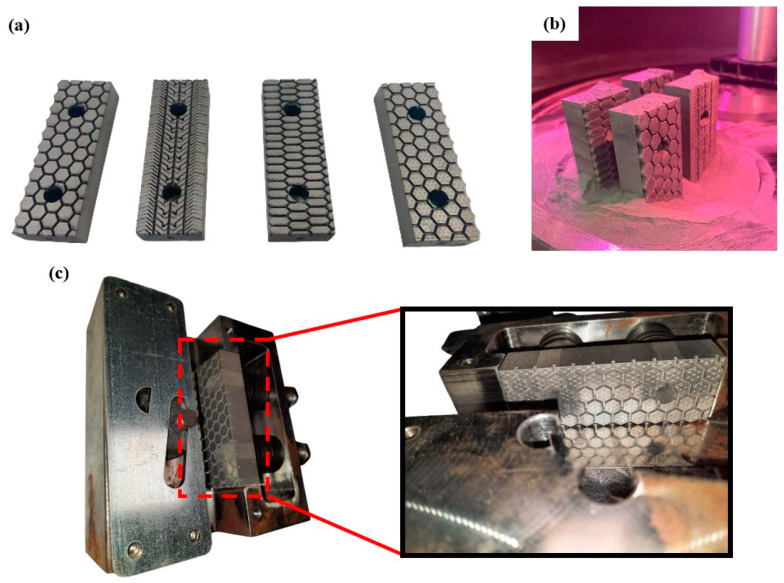
(**a**) The developed 3D-printed friction pads; (**b**) The friction pads inside the building chamber; (**c**) Indicative images of the assembly between the developed friction pads and the elevator safety gear.

The developed friction pads with advanced surface geometry were additively manufactured via the SLM 3D printing technique, as shown in Figure 5a. The 3D printing process was performed in a low concentration of oxygen (<1%) to avoid oxidation phenomena. Moreover, all four designed friction pads were fitted on the 3D printer’s platform in ZYX orientation as presented in Figure 5b. The overall 3D printing process lasted around 13 h indicating the rapid fabrication nature of AM technology. It is worth mentioning that the 3D prints were structurally flawless, without any visible defects and voids. Furthermore, the 3D-printed specimens were weighted with a high-accuracy scale and their weight values were found close to nominal values revealing negligible porosity for the structures. Then, the 3D-printed friction pads underwent the post-processing treatment of support removal and sandblasting in order to be finished. Finally, the developed friction pads were assembled in the system of a safety gear showing sufficient fit and functionality, as illustrated in Figure 5c.

### 3.2. Tribological Results

**Figure 6:**
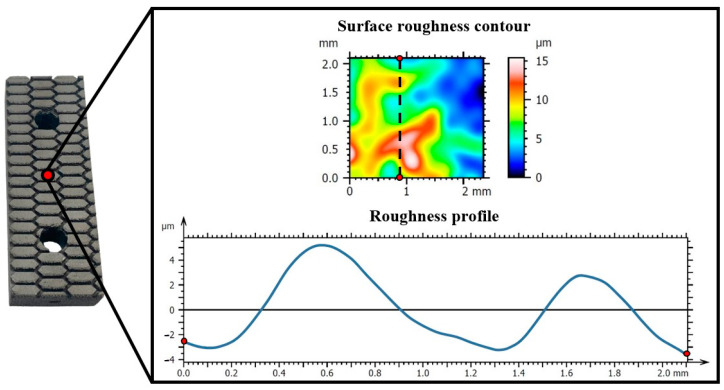
Surface roughness contour and roughness profile of an indicative braking surface of the 3D-printed pads.

Before the tribological analysis occurs, the roughness of the 3D-printed friction pads needs to be evaluated as it is strongly related to the CoF. The roughness measurements were performed on the contact surface of each specimen. Figure 6 illustrates an indicative example of the roughness evaluation on the 3D-printed parts. In detail, with the aid of the optical profilometer, both the surface roughness contour and roughness profiles were obtained. These acquired data show that the 3D-printed components in the examined plane (i.e., ΧΖ plane) have roughness fluctuating between 0 and 15 μm with smooth transitions.

It is worth noting that the examined area for roughness is independent of the patterns as it is an order of magnitude smaller (around 2 × 2 mm). Therefore, the 3D-printed surface was measured in roughness analysis which has similar value roughness for all developed braking pads due to the repeatability of the SLM 3D printing process. In addition, Table 2 lists all the crucial roughness parameters, i.e., average roughness (*S_a_, R_a_*), the root means square of the ordinate values (*S_q_, R_q_*), the mean roughness depth (*S_z_, R_z_*), the maximum pit height (*S_v_, R_v_*), and the maximum peak height (*S_p_, R_p_*).

**Figure 7:**
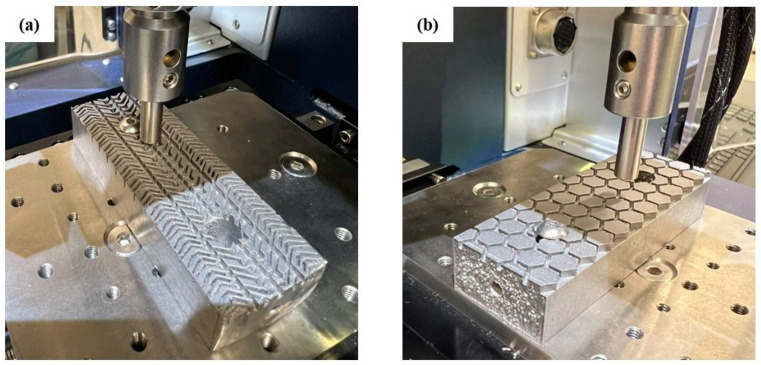
Indicative images of the (**a**) car-tire-like and (**b**) honeycomb friction pads during the pin-on-disc tribological tests.

After the roughness analysis of the 3D-printed surface of the developed friction pads, the next step was the conduction of tribological analysis. All specimens were examined under the same conditions and indicative images from this process are illustrated in Figure 7 for the car-tire-like pad (a) and for the honeycomb pad (b). It is obvious that the direction of the probe is the same as the elevator movement direction in order to obtain relatable tribological results.

**Figure 8:**
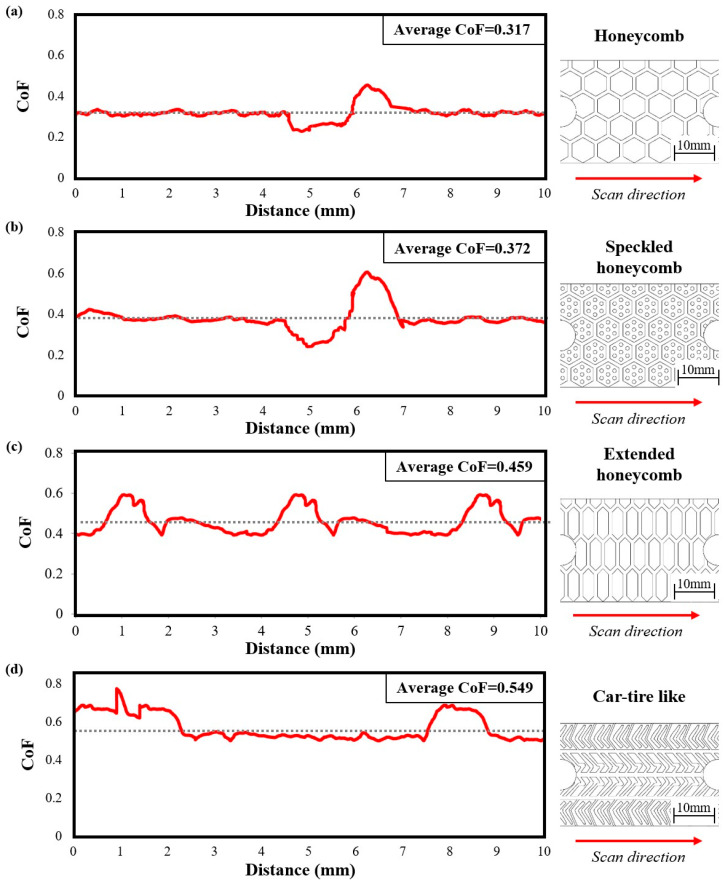
Tribological results of CoF to distance for pad’s structures of: (**a**) Honeycomb; (**b**) Speckled honeycomb; (**c**) Extended honeycomb, and (**d**) Car-tire-like.

Based on the tribological analysis, the honeycomb and honeycomb speckled configurations exhibited coefficients of friction (CoF) of 0.317 and 0.372, respectively. Remarkably, these values suggest a nearly identical pattern in their tribological behavior, owing to their closely matched CoF. However, the speckled variant displayed a slightly elevated CoF, likely attributable to the presence of speckles on its surface. This subtle distinction underscores the influence of surface texture on frictional dynamics, accentuating the importance of detailed analysis in tribological investigations. In the length of the sliding distance, we discern an excitation in Figure 8 due to the gap among the cells. For the extended honeycomb configuration, three excitations were observed, with the maximum CoF reaching 0.6. Additionally, the average CoF remained notably high, approximately around 0.459. In the car-tire-like configuration, the CoF is notably higher, reaching 0.549. This marked increase in CoF value highlighted the unique characteristics of the car-tire-like pattern, wherein its structural intricacies likely contributed to heightened frictional interaction. The car-tire-like pattern demonstrates the highest CoF, surpassing all the other configurations, solidifying its position as the configuration with the highest frictional propensity. This distinction underscores the diverse tribological behaviors manifested across different configurations, each bearing its own signature of frictional response.

### 3.3. Finite Element Analyses

After the conduction of tribological analyses, the obtained results, notably the CoF, were used to accurately simulate the mechanical performance of each designed friction pad under realistic loads. Therefore, utilizing the measured CoF for each design, the corresponding vertical force was evaluated, as listed in Table 3. The evaluation process was performed based on the biggest standardized elevator system with *P* + *Q* = 2600 kg according to Equations (1)–(3). It is obvious that the necessary vertical force that should be applied in order to decelerate the elevator and eventually stop it is strongly related to the interface CoF. In particular, when the CoF at the interface is low, it necessitates an increase in vertical force exerted in the opposite direction stressing the braking pad.

**Figure 9:**
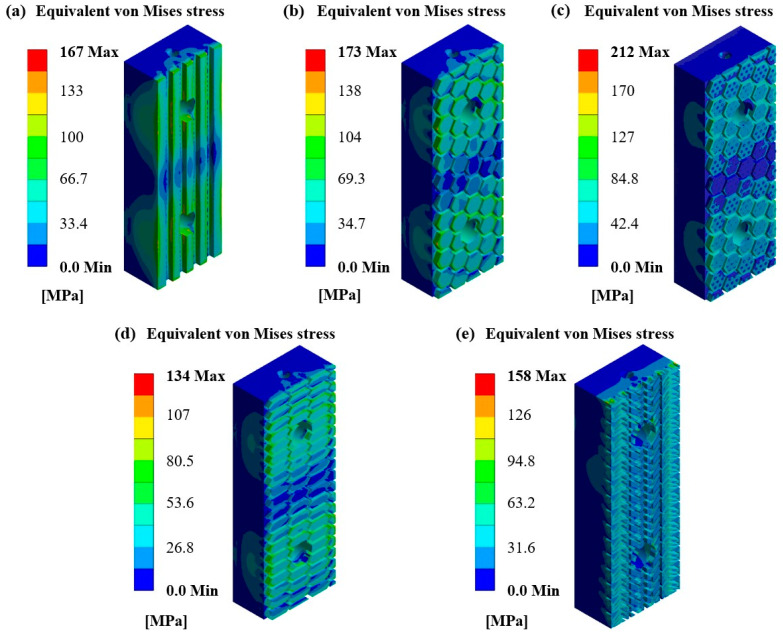
Equivalent von Mises contours for pad structures of: (**a**) Standard (**b**) Honeycomb; (**c**) Speckled honeycomb; (**d**) Extended honeycomb, and (**e**) Car-tire-like.

This observed behavior can easily result in the conclusion that the friction pads with low CoF are stressed more than the ones with higher CoF, leading to faster and abrupt part failure. Indeed, the conducted FEAs have shown similar results, as presented in Figure 9. More specifically, friction pads with the structures of extended honeycombs (Figure 9d) and car-tire-like (Figure 9e) exhibited lower stress concentrations, uniformly distributing the applied loads. In addition, the commercial friction pad (Figure 9a) and the friction pad with honeycomb structure (Figure 9b) revealed similar mechanical performance in terms of equivalent von Mises stress concertation showing a maximum von Mises stress value of around 170 MPa. On the other hand, the friction pad with speckled honeycomb structure (Figure 9c) concentrated the highest stress reaching 212 MPa, due to the localized high stress concentration regions derived from its honeycomb spots. It is worth noting that the main stress concentration regions are observed around the support holes of the part, indicating the increased applied loads on the bolts/screws. Finally, it is clear from the numerically extracted mechanical behavior of the designed friction pads that the metal 3D-printed structures are capable of withstanding the friction loads providing promising results for further experimental examination. In conclusion, the higher coefficient of friction configurations exhibited optimal stress distribution, thereby preventing abrupt failure and ensuring more uniform load distribution across braking pads, as revealed by the conducted FEAs.

## 4. Conclusions

In the current study, elevator progressive safety gear pads with bio-inspired contact surface structures were designed and developed in order to increase their durability and efficiency. More specifically, four different friction pads with embedded bio-inspired surface lattice structures were designed on the template of the conventional pad, namely the honeycomb, speckled honeycomb, extended honeycomb, and car-tire-like structures. Then, these designs were additively manufactured utilizing the SLM 3D printing technique in order to assess their compatibility with the existing system and experimentally evaluate their tribological behavior. The results revealed that the pads with high contact surface area, i.e., car-tire-like and extended honeycomb structure, possess high CoF with 0.549 and 0.459, respectively. Moreover, in the context of this paper, FEMs were developed according to the tribological behavior of each designed friction pad. The results of the numerical analyses showed that the friction pads with high CoF exhibit lower stress, increasing the durability and efficiency of the part. In addition, all designs were compared with the conventional friction pad and revealed superior performance. Then, through the conducted FEA, the developed friction pads experienced significantly lower stress than the construction material strength paving the way for further experimental evaluation through actual application to elevator progressive safety gear. To conclude, it is expected that the reduced loads and stress that the new designs exhibited will increase the number of uses of the braking pads, increasing their life cycle and reducing the maintenance cost and downtime of an elevator system. In addition, the uniform distribution of the stresses deriving from the applied braking force indicates smoother contact between the pads and the guide rails mitigating the risk of deforming them.

## 5. Patents

The research study was filled on 22 November 2023 in a patent application to the Hellenic Industrial Property Organization (HIPA) with the application number) 20230100969 and the title: «Hierarchical Biomimetic Surface Pattern on Elevator Friction Pads for Enhanced Dynamic Friction Performance».

## Figures and Tables

**Table 1:** PSD, Hall Flow, and Chemical Composition for H13 tool steel powder according to manufacturer.

Properties				Values		
Density				7.80 g/cm^3^		
Elastic modulus				200,000 ± 5000 MPa		
Poisson ratio				0.29		
Yield Strength (ASTM E8) [13]				1538 ± 31 MPa		
Ultimate Tensile Strength (ASTM E8) [13]				1903 ± 12 MPa		
Elongation at break (ASTM E8) [13]				11 ± 1 %		
**Nominal Range (μm)**		**D90 (μm)**		**D50 (μm)**	**D10 (μm)**	**Hall Flow (s/50 g)**
−45 + 15		50		34	21	≤25
**Weight Percent (nominal)**						
Fe	Cr		Mo	Si	V	C
Balance	5.2		1.3	1.0	1.0	0.4
**3D Printing Parameters**				**Values**		
Layer height				0.25 μm		
Hatching distance				0.40 μm		
Laser power				150 W		
Print speed				1000 mm/s		
VED				150 J/mm^3^		
Beam diameter				0.40 μm		

**Table 2:** Mean values of roughness indicators for the 3D-printed braking pads.

Surface Roughness		Profile Roughness	
Indicators	Values (μm)	Indicators	Values (μm)
*S_a_*	2.675 ± 0.1	*R_a_*	0.7081 ± 0.05
*S_q_*	3.168 ± 0.1	*R_q_*	0.8189 ± 0.05
*S_z_*	15.42 ± 0.4	*R_z_*	2.619 ± 0.1
*S_v_*	6.860 ± 0.2	*R_v_*	1.113 ± 0.1
*S_p_*	8.557 ± 0.25	*R_p_*	1.506 ± 0.1

**Table 3:** Vertical force evaluation based on CoF and necessary braking force for each structure.

Structure	Measured CoF	Braking Force (BF)	Vertical Force (VF)
Commercial	0.3	25,506 N	85,020 N
Honeycomb	0.317 ± 0.03		80,461 N
Speckled honeycomb	0.372 ± 0.04		68,565 N
Extended honeycomb	0.459 ± 0.05		55,569 N
Car-tire-like	0.549 ± 0.05		46,459 N

## Data Availability

The raw data supporting the conclusions of this article will be made available by the authors on request.
